# Quantitative assessment of the associations between ABCA1 gene polymorphism and glaucoma risk, evidence from a meta-analysis

**DOI:** 10.1097/MD.0000000000040427

**Published:** 2024-11-29

**Authors:** Fabin Wang, Xin Gou, Fan Wu, Hong Qiao, Dengli Zhao

**Affiliations:** a Department of Ophthalmology, Chong Gang General Hospital, Chongqing, China.

**Keywords:** ABCA1 gene, genetic susceptibility, glaucoma, meta-analysis, polymorphism

## Abstract

**Background:**

The association between polymorphisms in the ATP-binding cassette transporter A1 (ABCA1) gene and the risk of developing glaucoma has yielded conflicting results across various studies. This meta-analysis aims to comprehensively assess whether genetic variations in ABCA1 significantly contribute to the susceptibility to glaucoma.

**Methods:**

An extensive search was conducted across major databases, including PubMed, EMBASE, and the China National Knowledge Infrastructure (CNKI), covering all publications from the inception of each database through December 2023. Odds ratios (ORs) with 95% confidence intervals (CIs) were calculated to quantify the strength of the association between ABCA1 polymorphisms and glaucoma risk.

**Results:**

A significant association was observed between ABCA1 gene polymorphisms and glaucoma risk in the overall analysis, as demonstrated by allele contrast (*P* < .001), homozygote comparison (*P* < .001), heterozygote comparison (*P* < .001), recessive genetic model (*P* = .017), and dominant genetic model (*P* < .001). Notably, these associations were particularly pronounced in the Asian population, with all models showing statistical significance (*P* < .05). However, no significant association was detected in Caucasian or mixed populations, suggesting a potential ethnic specificity in the genetic susceptibility to glaucoma conferred by ABCA1 polymorphisms.

**Conclusions:**

Our findings indicate that ABCA1 polymorphisms may play a role in increasing the risk of glaucoma, specifically within Asian populations. This contrast highlights the importance of considering ethnic background in genetic association studies.

## 1. Introduction

Glaucoma, a complex optic neuropathy, is principally characterized by progressive thinning of the retinal nerve fiber layer (RNFL) and accompanying loss of visual field. Globally recognized as a leading cause of irreversible blindness, glaucoma stands as the second most prevalent eye disease, inflicting significant visual impairment and carrying a considerable social burden.^[1]^ Epidemiological studies estimate that nearly 64 million individuals across the globe are affected by glaucoma, with a prevalence rate of approximately 3.5% among the demographic aged 40 to 80 years. Given the demographic shifts towards an aging global population, projections suggest that the patient count could escalate to 111.8 million by the year 2040, underscoring the growing public health challenge posed by this condition.^[2]^

The risk factors for glaucoma are multifaceted, encompassing physiological changes such as slow anterior chamber angle-closure, zonular ligament laxity, and complications arising from antiglaucoma surgical interventions, alongside dramatic fluctuations in intraocular pressure. Beyond these physiological and procedural risk factors, a substantial body of evidence suggests a genetic predisposition to glaucoma, highlighted by the significant role of single nucleotide polymorphisms (SNPs). Over the past decade, the advent of genome-wide association studies (GWAS) and insights from mouse models have shed light on the genetic underpinnings of glaucoma, with particular emphasis on the association between the ABCA1 gene and the disease. Despite these advances, studies delving into the relationship between ABCA1 gene polymorphisms and susceptibility to glaucoma have yielded inconsistent and often conflicting results.

This inconsistency across various studies necessitates a comprehensive examination of the available evidence to discern the potential genetic association between ABCA1 polymorphisms and glaucoma risk. Therefore, the current study aims to consolidate and analyze the disparate findings through a systematic meta-analysis, endeavoring to clarify the role of ABCA1 polymorphisms in glaucoma susceptibility and contribute to the broader understanding of its genetic etiology. By aggregating data from a wide array of studies, this meta-analysis seeks to provide a more definitive stance on the connection between ABCA1 gene variations and the risk of developing glaucoma, particularly in diverse populations worldwide.

## 2. Materials and methods

### 2.1. Data collection strategy

A comprehensive literature search was conducted across several databases including PubMed, EMBASE, the Cochrane Library, Google Scholar, and Chinese National Knowledge Infrastructure (CNKI) to identify relevant studies. This search, conducted by 2 independent researchers, spanned from the inception of each database through to February 2024. The search terms employed were “polymorphism,” “glaucoma,” and “ABCA1,” tailored to each database’s specific requirements. This search was limited to studies published in English and Chinese to ensure a broad yet manageable scope of research for analysis.

### 2.2. Selection criteria for study inclusion and exclusion

Studies were selected based on the following inclusion criteria: case-control studies investigating the association between ABCA1 gene polymorphism and the risk of glaucoma; studies providing sufficient data to calculate odds ratios (ORs) and 95% confidence intervals (CIs). Exclusion criteria were applied to: studies not employing a case-control design; studies lacking adequate data for OR and 95% CI calculation; research focusing on animal subjects.

### 2.3. Data extraction and quality assessment

The 2 researchers independently extracted data and assessed the methodological quality of the included studies, adhering strictly to the predetermined inclusion and exclusion criteria. In cases of disagreement, a consensus was reached through discussion with a third, senior author. Given the nature of this meta-analysis, which synthesizes existing study data rather than involving direct human or animal subjects, the need for ethics approval and patient consent was deemed not applicable by the Ethics Committee.

### 2.4. Statistical analysis

Genotypic and allelic distributions of the ABCA1 polymorphism among cases and controls were analyzed using the chi-square (χ^2^) test. The Hardy–Weinberg equilibrium (HWE) for control group distributions was assessed using the chi-square test. Logistic regression models were utilized to compute ORs and 95% CIs, facilitating an evaluation of the association strength between ABCA1 polymorphism and glaucoma risk. Heterogeneity among studies was quantified using the *Q*-statistic and *I*^2^ statistics, guiding the choice between fixed-effects and random-effects models based on the observed heterogeneity levels.^[3]^ Five genetic models, as reported in previous literature, were examined to assess the association comprehensively.^[4,5]^ Sensitivity analysis and publication bias assessment were conducted in alignment with established meta-analytic practices.^[6–19]^ All statistical analyses were performed using Stata software, version 15.0, and this meta-analysis adheres to the PRISMA 2009 guidelines.^[20]^

### 2.5. Ethics and dissemination

Given the retrospective nature of the current study, the requirement for ethics approval was waived.

## 3. Results

### 3.1. Overview of study selection

**Figure 1.**
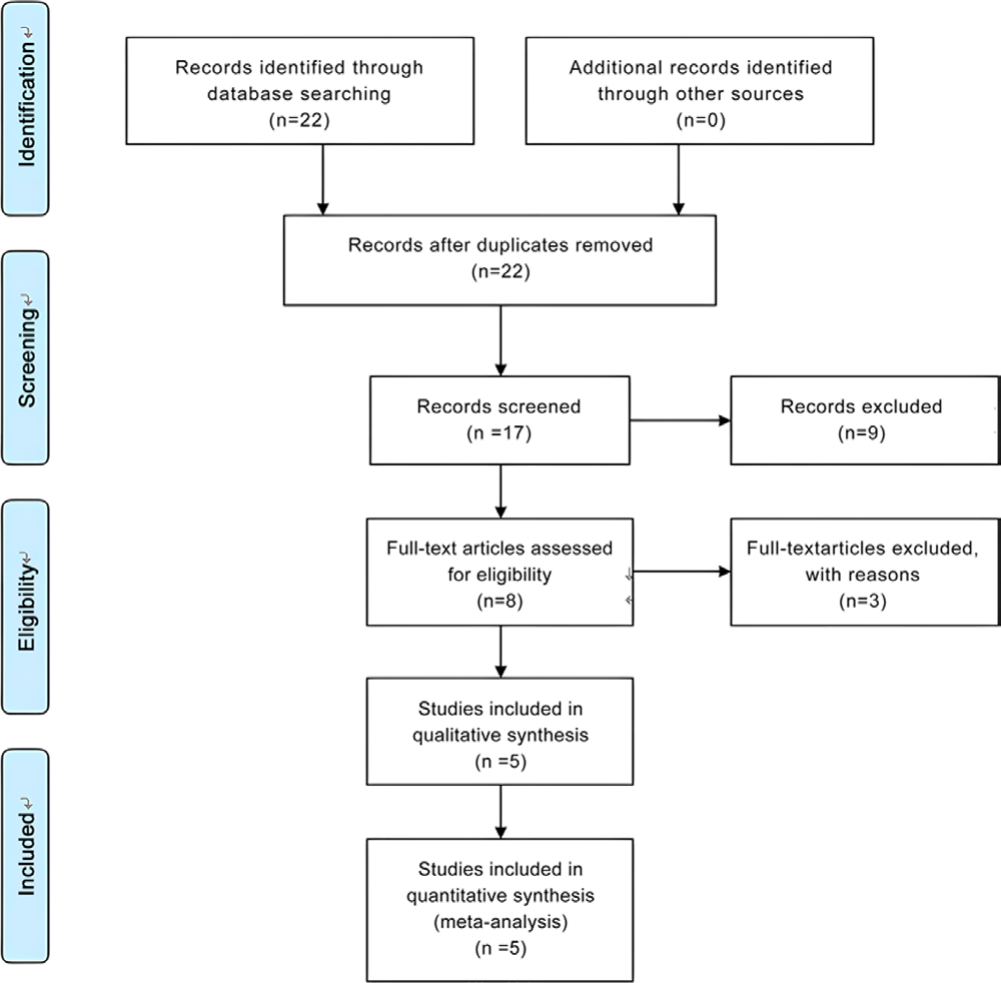
PRISMA 2009 flow diagram.

Following the Preferred Reporting Items for Systematic Reviews and Meta-Analyses (PRISMA) 2009 guidelines, our meta-analysis search process was systematically depicted in a PRISMA 2009 flow diagram (Fig. 1). Our rigorous selection process ultimately identified 6 studies eligible for inclusion in this comprehensive meta-analysis. The primary characteristics and data extracted from each study are meticulously summarized in Table 1, providing a clear overview of the evidence base. And Table 2 shows the quality assessment of the 7 case-control studies according to the Newcastle-Ottawa Scale.

**Table 1:** General information of eligible studies enrolled in the meta-analysis.

Ethnics (Country)	Genotyping method	Control origin	Sample capacity	Matching standard	HWE conformity	NOS
Caucasian (Saudi Arabia)	Taqman assays	HB	151/245	Age, sex, ethnicity	Yes	9
Mixed (Brazil)	Taqman assays	HB	505/504	Age, sex, ethnicity	Yes	8
Asian (China)	SNaPshotd	HB	1119/1303	Age, sex, ethnicity	Yes	8
Caucasian (Saudi Arabia)	Taqman assays	HB	100/245	Age, sex, ethnicity	Yes	9
Asian (China)	PCR-RFLP	HB	8552/7420	Age, sex, ethnicity	Yes	8
Asian (China)	PCR-RFLP	HB	1007/1009	Age, sex, ethnicity	Yes	8

Abbreviations: HB = hospital-based, HWE = Hardy–Weinberg equilibrium, NOS = Newcastle-Ottawa Scale.

**Table 2:** Quality assessment of the 7 case-control studies according to the Newcastle-Ottawa Scale.

Literature	Selection of enrolled study subjects	Between-group comparability	Exposure outcomes and factors	Total
Kondkar (2023)^[21]^	2	3	3	8
Araki (2022)^[22]^	3	3	3	9
Luo (2015)^[23]^	3	3	2	8
Kondkar (2022)^[24]^	2	3	3	8
Huang (2019)^[25]^	3	3	3	9
Chen (2014)^[26]^	3	3	3	9
Average	2.6	3.0	2.8	8.4

### 3.2. Association analysis between ABCA1 polymorphism and glaucoma risk

**Figure 2.**
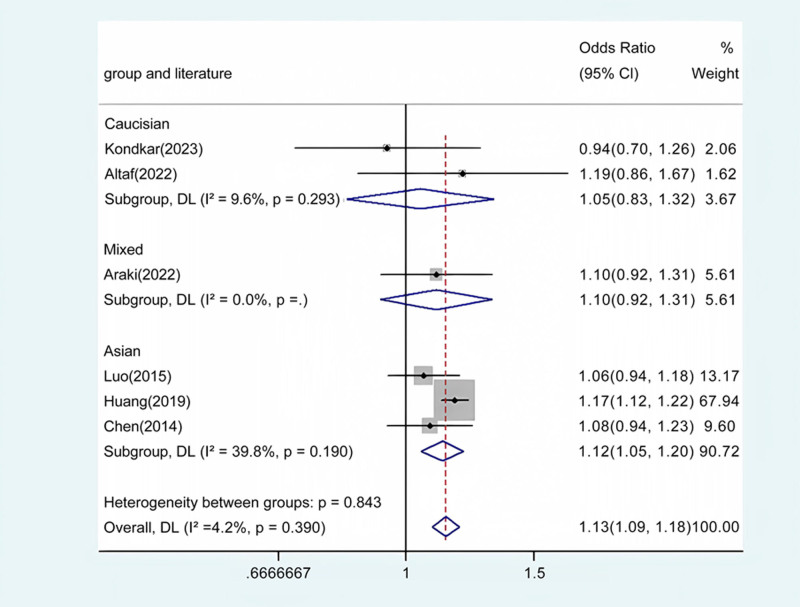
Forest plot for the associations between ABCA1 Polymorphism and glaucoma risk through allele contrast (G vs A). OR, odds ratio; CI, confidence interval.

**Figure 3.**
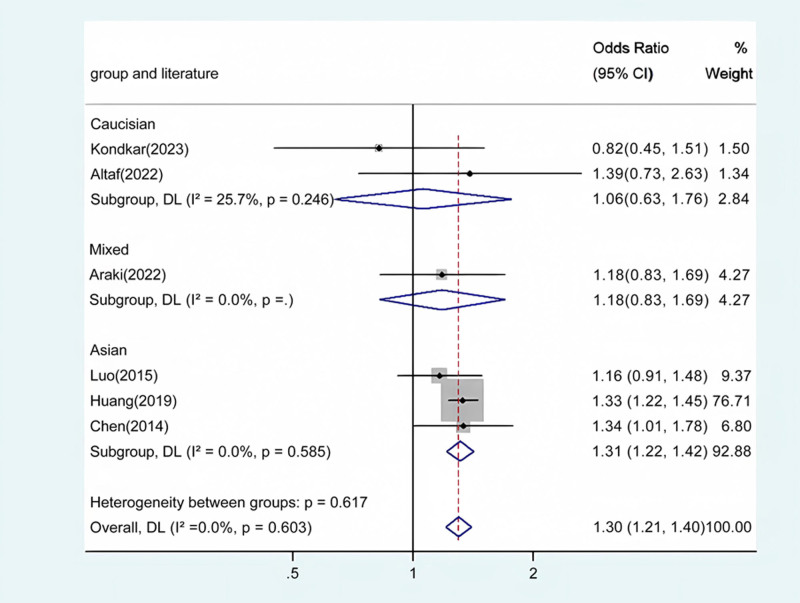
Forest plot for the associations between ABCA1 Polymorphism and glaucoma risk through homozygote comparison (GG vs AA). OR, odds ratio; CI, confidence interval.

**Figure 4.**
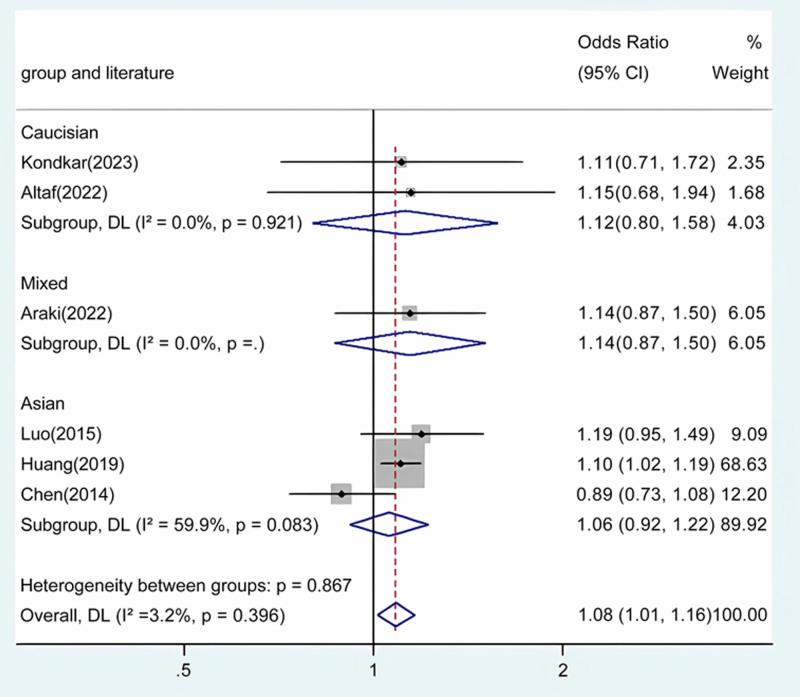
Forest plot for the associations between ABCA1 Polymorphism and glaucoma risk through heterozygosis comparison (GA vs AA). OR, odds ratio; CI, confidence interval.

**Figure 5.**
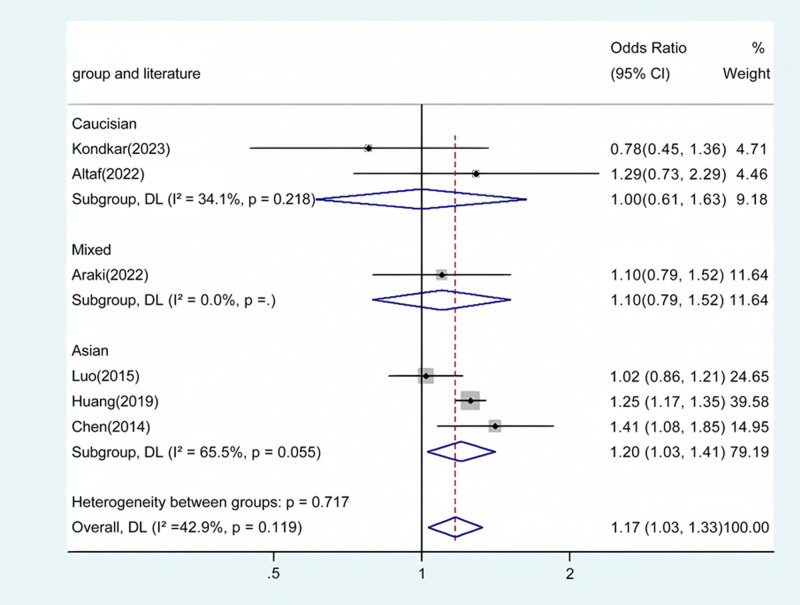
Forest plot for the associations between ABCA1 Polymorphism and glaucoma risk through recessive genetic model (GG vs GA/AA). OR, odds ratio; CI, confidence interval.

**Figure 6.**
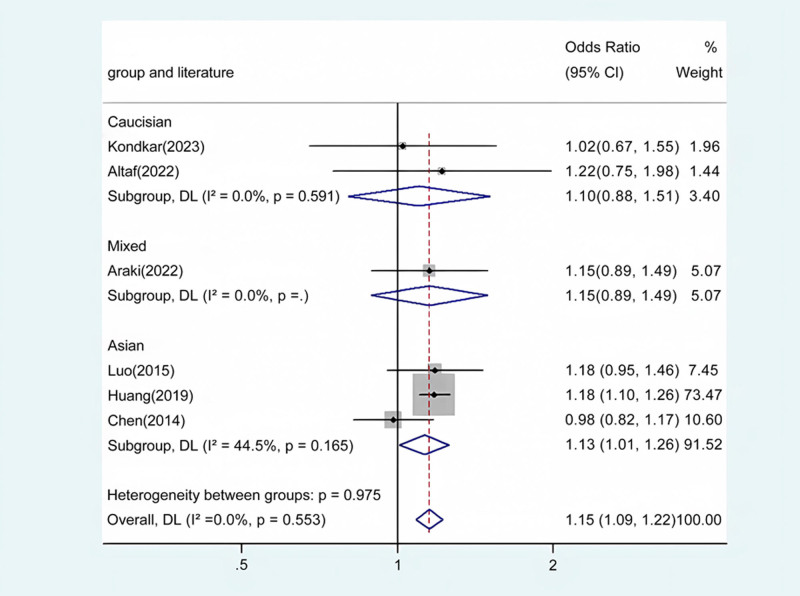
Forest plot for the associations between ABCA1 Polymorphism and glaucoma risk through dominate genetic model (GA/GG vs AA). OR, odds ratio; CI, confidence interval.

Our meta-analysis conducted a detailed evaluation across several genetic models to assess the relationship between ABCA1 polymorphism and glaucoma risk. Statistically significant associations were identified in the overall population across multiple comparative analyses: allele contrast revealed a strong association (*P* < .001) (Fig. 2), as did homozygote comparison (*P* < .001) (Fig. 3), heterozygote comparison (*P* < .001) (Fig. 4), recessive genetic model (*P* = .017) (Fig. 5), and dominant genetic model (*P* < .001) (Fig. 6). Notably, similar patterns of association were observed within the Asian demographic subgroup. The main findings highlighting the link between ABCA1 polymorphism and an increased risk of glaucoma are detailed in Table 3, underscoring the genetic influence on susceptibility to this eye condition.

**Table 3:** Meta-analysis of the ABCA1 rs2472493 polymorphism and PAOG risk.

Comparison	Population	N	Test of association			Mode	Test of heterogeneity		
			OR	95% CI	*P*		χ2	*P*	*I* ^2^
G vs A	Overall	6	1.13	1.09 to 1.18	<.001	Fixed	5.22	.390	4.2
	Caucasian	2	1.05	0.83 to 1.32	.701	Fixed	3.32	.293	9.6
	Asian	3	1.12	1.05 to 1.20	.001	Fixed	0	.190	39.8
	Mixed	1	0.83	0.55 to 1.26	.294	/	/	/	/
GG vs AA	Overall	6	1.30	1.21 to 1.40	<.001	Fixed	3.64	.603	0
	Caucasian	2	1.06	0.63 to 1.76	.836	Fixed	1.35	.246	25.7
	Asian	3	1.31	1.22 to 1.42	<.001	Fixed	1.07	.585	0
	Mixed	1	1.18	0.83 to 1.69	.360	/	/	/	/
GA vs AA	Overall	6	1.08	1.01 to 1.16	.020	Fixed	5.16	.396	3.2
	Caucasian	2	1.12	0.80 to 1.58	.503	Fixed	0.01	.921	0
	Asian	3	1.06	0.92 to 1.22	.445	Random	4.98	.083	59.9
	Mixed	1	1.14	0.87 to 1.50	.347	/	/	/	/
GG vs GA/AA	Overall	6	1.17	1.03 to 1.33	.017	Fixed	8.75	.119	42.9
	Caucasian	2	1.00	0.61 to 1.63	.090	Fixed	1.52	.218	34.1
	Asian	3	1.20	1.03 to 1.41	.023	Random	5.80	.055	65.5
	Mixed	1	1.10	0.79 to 1.52	.574	/	/	/	/
GG/GA vs AA	Overall	6	1.15	1.09 to 1.22	<.001	Fixed	3.98	.553	0
	Caucasian	2	1.10	0.80 to 1.51	.551	Fixed	0.29	.591	0
	Asian	3	1.13	1.01 to 1.26	.037	Fixed	3.61	.165	44.5
	Mixed	1	1.15	0.89 to 1.49	.282	/	/	/	/

Abbreviations: CI = confidence interval, OR = odds ratio, PAOG = primary open-angle glaucoma.

### 3.3. Heterogeneity assessment and meta-regression analysis

Our heterogeneity evaluation across the included studies revealed minimal variability in the overall population under all genetic models analyzed, indicating a consistent association across different studies. However, a moderate level of heterogeneity was noted in the Asian population subgroup, particularly in heterozygote comparison (*I*^2^ = 59.9) and recessive genetic model (*I*^2^ = 63.5). To explore the potential sources of this heterogeneity, we performed a meta-regression analysis. The findings suggested that the variation in sample sizes across studies contributed significantly to the observed heterogeneity, as detailed in the subgroup analysis results.

### 3.4. Sensitivity analysis and evaluation of publication bias

To verify the robustness of our findings, a sensitivity analysis was conducted. This analysis involved sequentially excluding each study to assess the impact on the overall meta-analysis outcomes. The results remained consistent regardless of the individual study excluded, demonstrating the stability and reliability of our conclusions. Additionally, publication bias was evaluated using Egger test, which did not reveal any significant asymmetry (*P* = .560), suggesting a low risk of bias in the published literature and supporting the validity of our meta-analysis results.

## 4. Discussion

Primary open-angle glaucoma (POAG) represents 1 of the leading causes of irreversible visual impairment globally, predominantly characterized by elevated intraocular pressure (IOP), distinctive optic nerve damage, and progressive visual field loss. The insidious nature of POAG often results in delayed diagnosis, as the absence of conspicuous clinical symptoms in the initial stages can lead to a missed opportunity for timely intervention. Emerging evidence suggests that the etiology of POAG is multifaceted, involving a complex interplay of genetic predispositions, systemic conditions such as hypertension, and ophthalmic characteristics, including alterations in the optic disc area and the vertical cup-to-disc ratio. These changes in ocular anatomy precede the deterioration of the visual field and exhibit a notable hereditary pattern. Epidemiological data underscore the heightened vulnerability within familial clusters, where the prevalence of POAG is reported to be 6-fold higher than in the broader population.

In the Asian demographic, particularly, the prevalence of primary angle-closure glaucoma (PACG) surpasses that observed in Western populations, a disparity that may be attributed to distinct anatomical features prevalent among individuals of Asian descent, such as shorter axial length and shallower anterior chamber depths. Additionally, variations in diagnostic standards between different regions further contribute to this discrepancy.^[27]^ Age remains a significant risk factor, with the majority of PACG cases diagnosed between the ages of 50 and 70.^[28]^ The pathogenesis of glaucoma is inherently complex, driven by a synergy of mechanical stress, genetic factors, immune responses, and anatomical predispositions. Specifically, the characteristic elevated IOP observed in PACG patients can exacerbate the production of glutamate, pro-inflammatory cytokines, and reactive oxygen species in the vicinity of the optic nerve head, thereby triggering the cascade of events leading to glaucomatous damage.^[29]^

Our meta-analysis elucidates that the association between ABCA1 polymorphism and glaucoma susceptibility varies significantly across different ethnic groups. While no correlation was identified within Caucasian or mixed populations, a pronounced association emerged among Asian individuals, suggesting that the presence of the G allele, along with GG and GA genotypes, significantly escalates the risk of glaucoma in this cohort. This variation underscores the critical role of genetic diversity in modulating disease risk and highlights the challenges encountered in genetic association studies, where population-specific genetic architecture can profoundly influence outcomes. To our knowledge, this investigation is the inaugural meta-analysis to scrutinize the link between ABCA1 polymorphism and glaucoma, employing a comprehensive analytical approach.

Nevertheless, our study is not without its limitations. The scope of included studies, particularly those representing African and other ethnic groups, remains narrow, underscoring the need for a more inclusive and updated meta-analysis that encapsulates a wider array of populations. Furthermore, the analysis was constrained by the unavailability of data on potential confounders and the relatively modest sample size, which may limit the generalizability of our findings.

In conclusion, the relationship between ABCA1 polymorphism and glaucoma risk appears to be modulated by ethnic background, with a significant association observed in the Asian population but not in Caucasian or mixed groups. This finding accentuates the necessity for future research endeavors to delve deeper into this genetic association, with a particular focus on expanding the ethnic diversity of study cohorts to validate and refine our understanding of the genetic underpinnings of glaucoma.

## Acknowledgments

We acknowledge some students from Chong Gang General Hospital for kind support.

## Author contributions

**Conceptualization:** Fabin Wang.

**Formal analysis:** Xin Gou, Fan Wu.

**Investigation:** Xin Gou, Fan Wu, Hong Qiao, Dengli Zhao.

**Supervision:** Fabin Wang.

**Writing – original draft:** Xin Gou, Fan Wu.

**Writing – review & editing:** Fabin Wang.
